# Deciphering the microbiome shift during fermentation of medicinal plants

**DOI:** 10.1038/s41598-019-49799-2

**Published:** 2019-09-17

**Authors:** Martina Köberl, Sabine Erschen, Mohammad Etemadi, Richard Allen White, Tarek F. El-Arabi, Gabriele Berg

**Affiliations:** 1 Graz University of Technology, Institute of Environmental Biotechnology, Graz, Austria; 2 Washington State University, Department of Crop and Soil Sciences, Pullman, WA USA; 3 Washington State University, Department of Plant Pathology, Pullman, WA USA; 4 Ain Shams University, Faculty of Agriculture, Cairo, Egypt; 5 Heliopolis University, Biotechnology Laboratory, Cairo, Egypt

**Keywords:** Environmental microbiology, Microbial ecology, Microbiome

## Abstract

The importance of the human-microbiome relationship for positive health outcomes has become more apparent over the last decade. Influencing the gut microbiome via modification of diet represents a possibility of maintaining a healthy gut flora. Fermented food and lactic acid bacteria (LAB) display a preventive way to inhibit microbial dysbioses and diseases, but their ecology on plants is poorly understood. We characterized the microbiome of medicinal plants (*Matricaria chamomilla* L. and *Calendula officinalis* L.) using 16S rRNA gene profiling from leaves that were fermented over a six-week time course. The unfermented samples were characterized by a distinct phyllosphere microbiome, while the endosphere revealed a high similarity. During fermentation, significant microbial shifts were observed, whereby LAB were enhanced in all approaches but never numerically dominated. Among the LAB, *Enterococcaceae* were identified as the most dominant family in both plants. *M. chamomilla* community had higher relative abundances of *Lactobacillaceae* and *Carnobacteriaceae*, while *C. officinalis* showed a higher presence of *Leuconostocaceae* and *Streptococcaceae*. The natural leaf microbiome and the indigenous LAB communities of field-grown *Asteraceae* medicinal plants are plant-specific and habitat-specific and are subjected to significant shifts during fermentation. Leaf surfaces as well as leaf endospheres were identified as sources for biopreservative LAB.

## Introduction

In recent years, the crucial role of the microbiome for plant and human health has been further elucidated, in particular by the advent of next-generation sequencing^1^. Everyday more evidence is suggesting the role and potential of the microbiome to prevent disease^2^. However, as one of the first, Metchnikoff theorized already over 100 years ago that ‘*there is a dependence of the intestinal microbes and the food*’ and that these microorganisms can ‘*modify the flora of our bodies and to replace the harmful microbes by useful microbes*’. He stated even back then that especially lactic acid bacteria (LAB) have a positive effect when ingested: ‘*A reader who has little knowledge of such matters may be surprised by my recommendation to absorb large quantities of microbes, as the general belief is that microbes are all harmful. This belief, however, is erroneous. There are many useful microbes, amongst which the lactic bacilli have an honourable place*’^3^. Traditionally, fermented food was part of all known diets worldwide because it allowed the production and especially the preservation of tasty food. Besides yeasts, LAB are used to convert carbohydrate-containing substances in homofermentative or heterofermentative ways into lactic acid, their primary fermentation product. Since ancient time, the production of lactic acid during fermentation made LAB a vital tool to preserve, for example, milk and vegetables. Nowadays, this ancient fermentation process has become a more industrialized and sophisticated biotechnological process, where parameters no longer are left to chance. The use of selected starters does make fermentation a valuable method, ensuring a safe food end-product with enhanced properties regarding the sensory and nutritional characteristics, but also contributing to amended shelf life and guaranteeing repeatable quality^4,5^.

While sauerkraut (fermented cabbage) is still popular and distributed, many other fermented foods are lost from the modern western diet^6^. A similar trend is also noticeable for Asia, actually known for its healthy diet, where a shift from traditional food towards commercial fast food in combination with urbanization has led to a decline in the consumption of fermented aliments^7^. In contrast, new food was designed by LAB application because of its health value, it’s nontoxic, and helps promote the natural intestinal biota as a probiotic^8–10^. In particular in Asia, some plants are fermented for medical applications. However, little is known about fermentation products of classical western medicinal plants, despite the frequent use of other forms of preservation (tincturing or drying). A study comparing native and fermented chamomile extracts^11^ revealed the maintenance of the chamomile’s antioxidant, antimicrobial and cytotoxic activity after fermentation. The authors further reported an increase of the bioactive flavonoid apigenin – also a promising compound in cancer research – in the fermented plant materials, indicating the effectiveness of the fermentation process responsible for the hydrolysis of its bound forms, which are generally attributed with lower bioactivity than the aglycone^11^. Park *et al*.^12^ even recorded improved antioxidative and cytotoxic activities of chamomile florets fermented by *Lactobacillus plantarum* KCCM 11613P. Green tea combined with leaves of *Houttuynia cordata* Thunb. fermented using *Lactobacillus paracasei* subsp. *paracasei* NTU 101 resulted in a product with anti-adipogenic and anti-obesity effects^13^. These effects could mainly be attributed to increased levels of the polyphenolic compounds epigallocatechin gallate (EGCG) and epigallocatechin (ECG), as well as chlorogenic acid, which were formed in the early stage of the fermentation. Compared to the non-fermented tea, the fermented product was able to stimulate lipolysis combined with a decrease of body weight gain and body fat pad^13^. Besides their bioactive secondary metabolites, medicinal plants are characterized by a unique profile of naturally associated microorganisms^14^, and we hypothesize that these indigenous microbial inhabitants form a unique fermented product with an extraordinary and specific fermentation microbiome.

Several LAB are used as probiotics defined as ‘*live microorganisms which when administered in adequate amounts confer a health benefit on the host*’^15^. Health benefits derived through ingestion of probiotics include cancer prevention, the regulation of cell proliferation and apoptosis^16^. Studies further reported an antagonistic potential of LAB due to their antimicrobial activities against a long list of human pathogens^17,18^. Moreover, studies confirmed the involvement of LAB in the inhibition of micelle formation in the intestine, which consequently results in positive effects on the serum cholesterol^19^. Also, LAB can reduce the onset of systemic inflammatory induced diabetes and enhance the lipid metabolism^20,21^. Today, we know that the composition of the gut microbiome plays a vital role in a wide range of host-related processes including human health^2,10^. The gut microbiome has been associated with the promotion of obesity^22,23^ or depression^24^. Probiotics, including their downstream metabolites, have been suggested to play a significant role in the formation and establishment of a well-balanced intestinal microbiota^25,26^. LAB can have several advantageous mechanisms that make them beneficial: (1) the ability to adhere to the cell, (2) reduce pathogenic bacteria adherents, (3) co-aggregate, (4) produce organic acids, hydrogen peroxide, bacteriocins, and other metabolites which antagonize pathogenic microorganisms. LAB are excellent candidates for modifying the human gut microbiome as a majority are nonpathogenic and directly colonize^25,26^.

Plants are a vital source of LAB but not systematically studied. The ambition of this study was the comparative examination of the phyllosphere microbiome of two well-known medicinal plants regarding their colonization with LAB. The major question was whether naturally occurring LAB can be enriched throughout a fermentation process of the plant material. Natural fermentation approaches from leaf material of organically grown medicinal plants, *Matricaria chamomilla* L. and *Calendula officinalis* L., were performed, with the aim to potentially detect new sources for probiotic and biopreservative LAB. Composition and diversity of the bacterial phyllosphere communities were investigated separately for the leaf ectospheres and endospheres, and the microbiome dynamics were followed over a six-week fermentation period. In parallel, to the fermentation of medicinal plants, comparative sauerkraut fermentations of *Brassica oleracea* var. *capitata* L. were performed. The culturable bacterial fraction was compared between plant species in terms of abundance and LAB diversity and taxonomy.

## Results

### The bacterial phyllosphere microbiome of *Matricaria chamomilla* L. and *Calendula officinalis* L. – the natural fermentation inoculum

**Figure 1:**
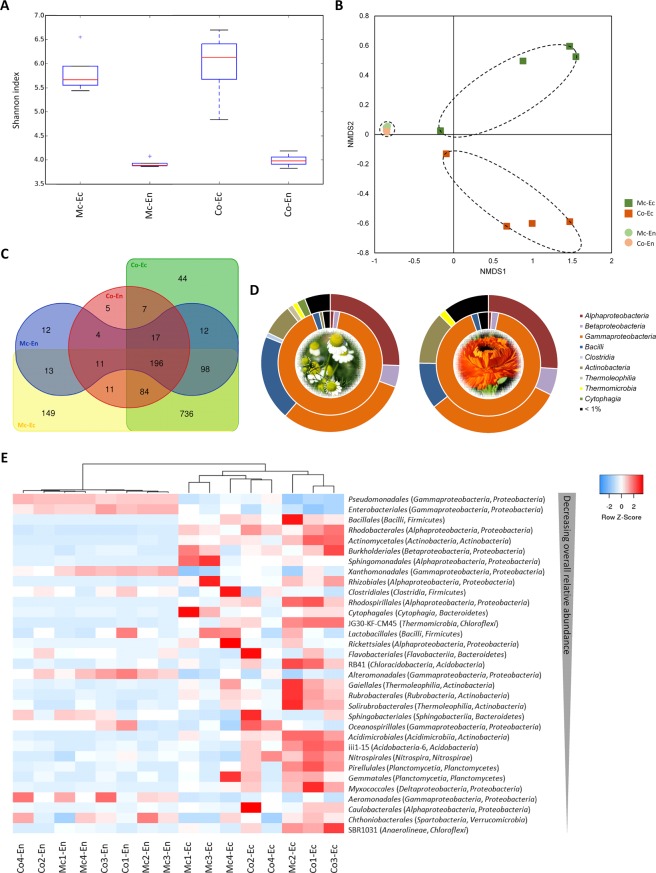
The bacterial phyllosphere microbiome colonizing the leaf ectosphere (Ec) and endosphere (En) of *Matricaria chamomilla* L. (Mc) and *Calendula officinalis* L. (Co) grown under desert-farming conditions in Egypt. (**A**) Shannon diversity at a genetic distance of 3%. (**B**) Nonmetric multidimensional scaling (NMDS) plot based on Bray-Curtis dissimilarities. The corresponding stress value is 0.02. (**C**) Venn diagram showing the distribution of bacterial OTUs across plant species and microenvironments. (**D**) Taxonomic composition at the class level. The outer circles represent the ectosphere colonization, while the inner circles show the class distribution within the leaf endophytes. (**E**) Heat map displaying the relative abundance of orders with over 0.1% of overall relative abundance in descending order. The dendrogram is based on average linkage clustering and Manhattan distances. (**A**–**E**) Data were ascertained by 16S rRNA gene profiling in four independent replicate samples per plant species.

To have a detailed view of the original phyllosphere microbiomes of *M. chamomilla* and *C. officinalis*, leaves were dissected for inner and outer compartments. Leaves were separated into outer surfaces (ectosphere) and inner tissues (endosphere). We obtained 1,399 bacterial operational taxonomic units (OTUs, 16S rRNA genes at 97% similarity), with up to 241 OTUs per ectosphere and 89 OTUs per rarefied endosphere sample. Alpha diversity (Shannon index) indicated that bacterial diversity in the leaf endosphere was significantly lower than in the ectosphere in both *M. chamomilla* and *C. officinalis* (Fig. 1A, Table S1, p < 0.05). The endospheres of *M. chamomilla* and *C. officinalis* had similar alpha diversity, whereas the ectosphere had more variability in *C. officinalis* over *M. chamomilla*. A nonmetric multidimensional scaling (NMDS) analysis based on the dissimilarity matrix calculated using the Bray-Curtis metric showed a clear separation of ectosphere and endosphere samples (Fig. 1B, p = 0.001). The endosphere samples of both plants were highly similar and clustered tightly together, while the ectosphere samples were separated but not statistically different. Nevertheless, this indicates that although the bacterial diversity in the ectosphere of the two plants was similar, they had a differing bacterial composition (Fig. 1C, 82 significantly different OTUs_0.97_).

The composition of the bacterial communities at phylum level was numerically dominated by *Proteobacteria*, *Firmicutes*, and *Actinobacteria*, comprising >90% of relative abundance in the ectosphere of both medicinal plants. Additionally, *Chloroflexi* and *Bacteroides* were found in both plant ectospheres in a relative abundance of over 2%. At the class level, *Alpha-*, *Beta-* and *Gammaproteobacteria*, *Bacilli*, and *Actinobacteria* were the major representative taxa in the ectosphere communities (Fig. 1D,E). The leaf endosphere of both plants was numerically dominated by *Proteobacteria* (~95% in both endosphere communities). *Firmicutes* and *Actinobacteria* were found in lower abundance in both endospheres (>1%). The *Proteobacteria* inhabiting the inner plant tissue were divided amongst the three bacterial classes *Alpha-*, *Beta-* and *Gammaproteobacteria*, whereby *Gammaproteobacteria* were highly predominant (~97% of classified *Proteobacteria* and ~91% of the total endophytic microbiome). *Bacilli* were detected at low abundance (~2%) within the endospheres. *Lactobacillales* were detected in the leaf ectosphere as well as endosphere of both medicinal plants (0.7 to 0.3%), whereby no clear habitat preference was discernible.

### General characterization and dynamic of bacterial communities during fermentation

**Figure 2:**
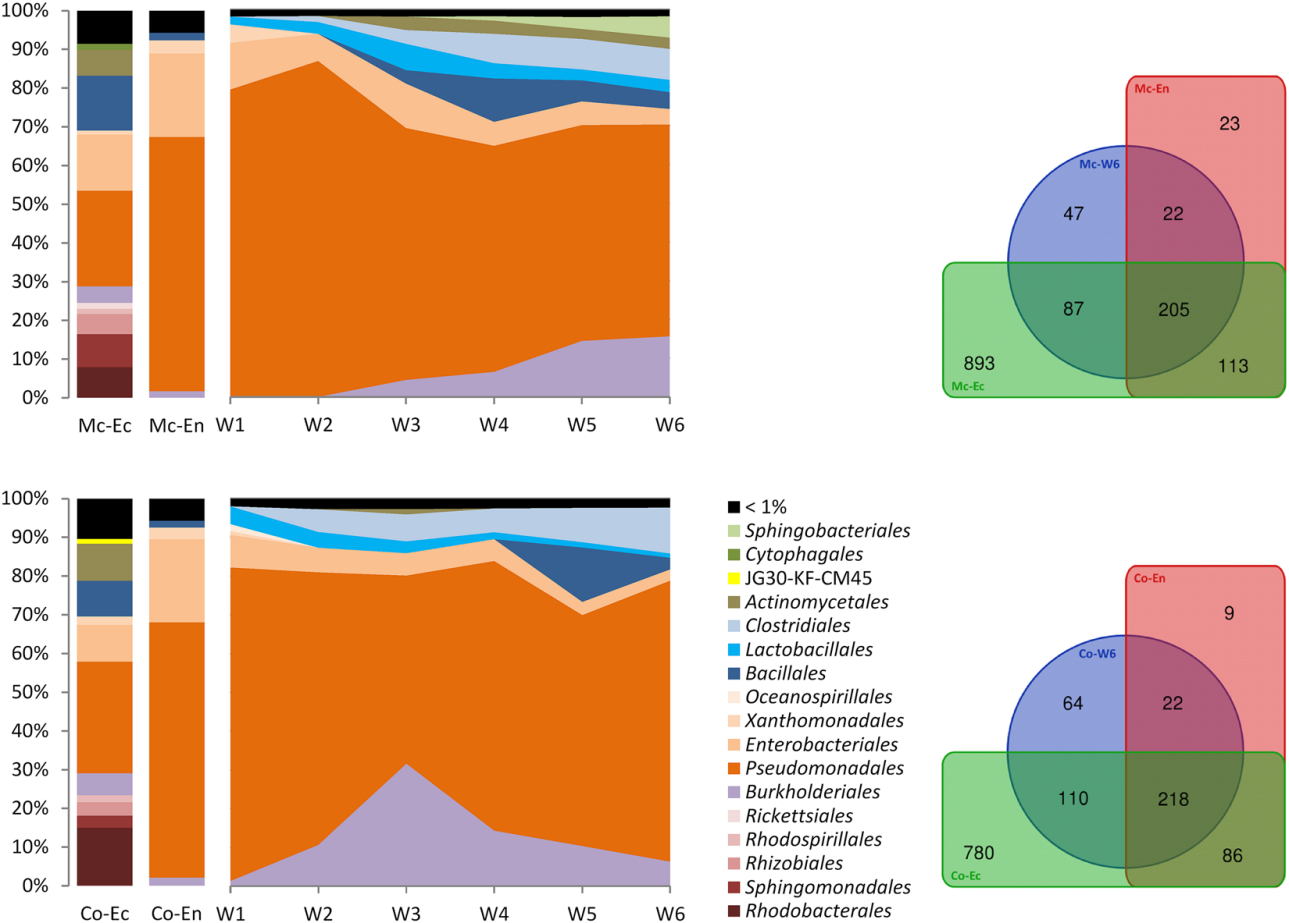
Order composition of the bacterial microbiome in fresh leaves of *Matricaria chamomilla* L. (Mc) and *Calendula officinalis* L. (Co) and dynamics over a six-week fermentation period (W1-W6). First two columns represent the ectospheric (Ec) and endospheric (En) leaf colonization. Mean values of four independent replicate samples subjected to 16S rRNA gene profiling are depicted for each plant species. Venn diagrams feature the bacterial OTU shift from original (Ec and En) to fermented leaves (W6).

Alpha diversity (Shannon index) in the fermentation approaches was significantly lower than in the original leaf ectospheres (Table S1, p = 0.003). Concerning the bacterial community composition in *M. chamomilla* and *C. officinalis* during six weeks of fermentation, the predominant phyla were *Proteobacteria* and *Firmicutes* (Fig. 2). Members of *Actinobacteria* and *Bacteroidetes* were more prevalent in *M. chamomilla* than in *C. officinalis*. Other phyla were represented in the fermentations at very low abundances (<0.1%), which were *Acidobacteria*, *Verrucomicrobia*, *Chloroflexi*, *Nitrospirae*, *Tenericutes*, *Planctomycetes*, *Gemmatimonadetes*, AD3, and *Thermi*. TM6 was only detected in the earlier stage (week 1 to 3) of the *Calendula* fermentation.

At the order level, the most numerically dominant taxa were *Pseudomonadales*, *Burkholderiales*, and *Enterobacteriales*, followed by *Clostridiales*, *Bacillales*, and *Lactobacillales* (Fig. 2). As a reflection of the microbial community dynamics, the relative abundances of bacterial OTUs varied between plant species and over the fermentation period. Almost all taxa which were detected in the fermentation approaches were also found in the freshly collected plant samples, but sometimes in quite subordinate presence, e.g., the LAB (<0.8% in the phyllosphere microbiomes). *Alphaproteobacteria* were identified as abundant members of the ectospheric leaf communities (26.1% to 25.6%) but were hardly detectable during fermentation (<0.7%). The relative abundance of *Firmicutes* (*Bacilli* and *Clostridia*) increased in both approaches over time, whereby *Lactobacillales* did not significantly change after the initial increase within the first week of fermentation. The relative abundance of *Actinomycetales* and *Sphingobacteriales* increased during the fermentation of *M. chamomilla* over time.

### Dynamic of lactic acid bacteria communities

**Figure 3:**
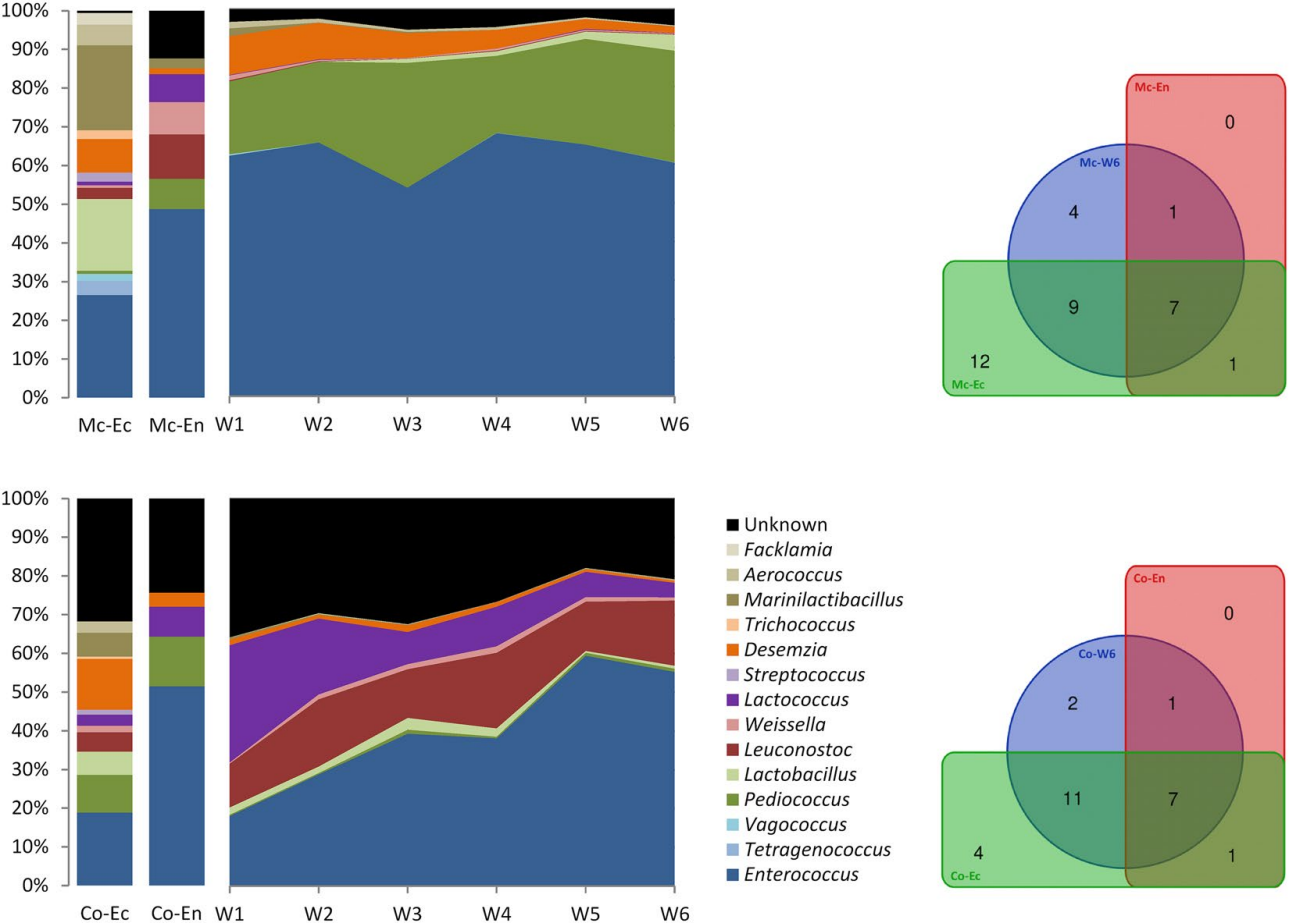
Genus structure of the *Lactobacillales* communities in fresh leaves of *Matricaria chamomilla* L. (Mc) and *Calendula officinalis* L. (Co) and dynamics over a six-week fermentation period (W1-W6). First two columns represent the ectospheric (Ec) and endospheric (En) leaf colonization. Mean values of four independent replicate samples subjected to 16S rRNA gene profiling are depicted for each plant species. Venn diagrams feature the lactic acid bacterial OTU shift from original (Ec and En) to fermented leaves (W6).

During *M. chamomilla* fermentation, *Lactobacillales* reached their highest relative abundance (6.8%) after three weeks of fermentation. Whereas during *C. officinalis* fermentation, *Lactobacillales* reached their maximum abundance (4.6%) after one week and gradually decreased throughout the fermentation (Fig. 2). Taxonomically, LAB could be affiliated to six families which included the *Enterococcaceae*, *Lactobacillaceae*, *Leuconostocaceae*, *Streptococcaceae*, *Carnobacteriaceae*, and *Aerococcaceae*. All families where found in original plant samples and throughout fermentation. *Enterococcus* (*Enterococcaceae* family) was the most numerically dominant genus during both medicinal plant fermentations. *Enterococcus* was highest after four weeks in the chamomile fermentation (67.9%) and after five weeks in the *Calendula* fermentation (59.5%) (Fig. 3). In the fresh leaves, *Enterococcus* accounted for 26.5% to 18.8% of the ectospheric LAB communities and nearly half (51.5% to 48.8%) of the endophytic LAB. However, one must consider that the presence of *Lactobacillales* within the total bacterial microbiome was much lower in the unfermented samples. Other members of *Enterococcaceae* (genera *Tetragenococcus* and *Vagococcus*) were exclusively detected in the ectosphere of the chamomile but disappeared during fermentation. *Pediococcus* belonging to the *Lactobacillaceae* was identified as abundant genus throughout the chamomile fermentation (max. 32.2% after three weeks), while it played a minor role in the *Calendula* fermentation (max. 1.0% after three weeks). *Pediococcus* (*Lactobacillaceae*) was detected as ectophyte and endophyte in both medicinal plants. *Lactobacillus* (*Lactobacillaceae*) was present in the leaf ectospheres and the fermentation approaches, reaching its highest relative abundance after six weeks of chamomile fermentation (4.2%). *Calendula* fermentation had a lower abundance of *Lactobacillus* (max. 3.0% after three weeks). While *Lactobacillaceae* were a numerically dominant group in the chamomile fermentation, *Leuconostocaceae* (genera *Leuconostoc* and *Weissella*) and *Streptococcaceae* (genus *Lactococcus*) were identified as abundant LAB in the fermentation of *Calendula*. *Streptococcus* (*Streptococcaceae*) was detected in both ectosphere communities but was not present in the endospheres and during fermentation. *Desemzia* belonging to the *Carnobacteriaceae* was the third abundant genus in the early fermentation of *M. chamomilla* diminishing over time, and had lower relative abundance but was stable throughout the *Calendula* fermentation. *Trichococcus* (*Carnobacteriaceae*) was detected in the leaf ectosphere of both plants, but not in the inner tissue and at <0.1% in the fermentation approaches. *Aerococcaceae* (genera *Marinilactibacillus*, *Aerococcus*, and *Facklamia*) were quite present in the original leaf samples (30.3% in the *M. chamomilla* ectosphere and 9.1% in the *C. officinalis* ectosphere) but revealed a subordinate presence within the course of fermentation.

### Cultivation and characterization of lactic acid bacteria

On R2A agar after aerobic incubation, colony forming units after six weeks of fermentation were highly similar in both medicinal plant species (*M. chamomilla* 8.25 ± 0.29 log_10_ CFU ml^−1^, *C. officinalis* 8.25 ± 0.01 log_10_ CFU ml^−1^). Slightly lower was the quantification after aerobic cultivation on R2A agar from the comparative sauerkraut fermentation of *Brassica oleracea* var. *capitata*: 6.95 ± 0.34 log_10_ CFU ml^−1^. On MRS agar, statistically significantly higher CFU counts were ascertained for the fermentation of *M. chamomilla* than for *C. officinalis* and *B. oleracea* (p < 0.05): *M. chamomilla*: 8.32 ± 0.23 log_10_ CFU ml^−1^, 8.39 ± 0.22 log_10_ CFU ml^−1^, and 8.14 ± 0.27 log_10_ CFU ml^−1^, for aerobic, anaerobic, and microaerobic conditions, respectively; *C. officinalis*: 7.26 ± 0.21 log_10_ CFU ml^−1^, 7.18 ± 0.13 log_10_ CFU ml^−1^, and 7.07 ± 0.24 log_10_ CFU ml^−1^, for aerobic, anaerobic, and microaerobic conditions, respectively; and *B. oleracea*: 7.00 ± 0.34 log_10_ CFU ml^−1^, 6.20 ± 1.22 log_10_ CFU ml^−1^, and 6.49 ± 0.68 log_10_ CFU ml^−1^, for aerobic, anaerobic, and microaerobic conditions, respectively.

Out of 324 isolates collected from the fermentation approaches of the medicinal plants (*M. chamomilla* 168, *C. officinalis* 156), 248 isolates were classified as LAB (*M. chamomilla* 141, *C. officinalis* 107) by physiological testing (Gram-positive and catalase-negative), genomic fingerprinting, and 16S rRNA gene sequencing. *B. oleracea* yielded a total of 234 isolates with 135 LAB. Of the LAB collection isolated from the medicinal plants, 101 LAB were obtained from microaerobic cultivation on MRS agar, 77 and 47 from aerobic and anaerobic incubation of MRS agar, respectively, and 23 isolates from aerobic cultivation on non-selective R2A agar. The selected cabbage isolates were more equally distributed between cultivation conditions: 38 isolates originated from microaerobic cultivation on MRS agar, 36 from aerobic cultivation on R2A agar, and 34 and 27 isolates were obtained from aerobic and anaerobic cultivation on MRS agar, respectively.

Using restriction fragment length polymorphism of the 16S rRNA gene, the LAB isolates obtained from the medicinal plants could be clustered into three groups comprising 172, 68 and eight medicinal plants isolates, respectively. Isolates from the larger two clusters were also obtained from the cabbage fermentation (one and 128 isolates, respectively), while the third cluster was exclusively associated with isolates from the medicinal plants. Two additional clusters comprising five and one isolates were found to be specific for the cabbage fermentation. A representative set of isolates with differing BOX-PCR genomic fingerprints covering all five taxonomic groups was selected for partial 16S rRNA gene sequencing. The LAB isolates could be phylogenetically assigned to two genera, namely *Enterococcus* and *Lactobacillus*. *Enterococcus* isolates belonging to the first cluster (154_CoS2-11, 233_CoF3-9, and 353_CoF4-1) had high sequence similarities to *E. casseliflavus* with 99% similarity (Table 1). Isolates of all the other clusters were identified as *Lactobacillus* spp. Isolates 212_McS3-12, 316_McF4-12, 218_CoS3-6, and 183_KGS3-10 of the taxonomic cluster 2 were most closely related to *L. plantarum*/*L. pentosus*, *L. brevis* and *L. coryniformis* with 99% similarity, respectively. Isolate 287_McS4-7 of the medicinal plant-specific cluster 3 showed the highest similarity to *L. nenjiangensis* with 99% of sequence similarity. The two cabbage-specific clusters were with 99% of sequence similarity most closely related to *L. paracasei* and *L. fabifermentans*, respectively. Isolates of each taxonomic cluster found for the medicinal plants’ fermentations (cluster 1, 2 and 3) were cultivated from the fermentation approaches of both medicinal plants. Two clusters of *Lactobacillus* were more dominant in the fermentation approaches of *M. chamomilla* (cluster 2: 64 isolates from *M. chamomilla* and four from *C. officinalis*; cluster 3: seven from *M. chamomilla* and one from *C. officinalis*), the *Enterococcus* cluster in those of *C. officinalis* (cluster 1: 70 from *M. chamomilla* and 102 from *C. officinalis*). In the *B. oleracea* fermentations, the *Lactobacillus* cluster 2 was the most frequently occurring, while just one single *Enterococcus* isolate (cluster 1) was obtained.

**Table 1:** Identification of selected LAB isolates according to 16S rRNA gene sequencing.

Isolate	Isolation source	Cultivation method	ARDRA cluster^a^	Closest database match (Accession number)^b^	Similarity	Sequence accession number^c^
154_CoS2-11	*C. officinalis* Sekem	MRS aerobic	1	*Enterococcus casseliflavus* (NR_119280.1)	99%	LR216271
233_CoF3-9	*C. officinalis* Faiyum	MRS anaerobic	1	*Enterococcus casseliflavus* (NR_119280.1)	99%	LR216272
353_CoF4-1	*C. officinalis* Faiyum	MRS microaerobic	1	*Enterococcus casseliflavus* (NR_104560.1)	99%	LR216273
212_McS3-12	*M. chamomilla* Sekem	MRS anaerobic	2	*Lactobacillus plantarum* (NR_104573.1) *Lactobacillus pentosus* (NR_029133.1)	99% 99%	LR216274
316_McF4-12	*M. chamomilla* Faiyum	MRS microaerobic	2	*Lactobacillus brevis* (NR_116238.1)	99%	LR216275
218_CoS3-6	*C. officinalis* Sekem	MRS anaerobic	2	*Lactobacillus coryniformis* (NR_029018.1)	99%	LR216276
183_KGS3-10	*B. oleracea* Sekem, big	MRS anaerobic	2	*Lactobacillus plantarum* (NR_104573.1) *Lactobacillus pentosus* (NR_029133.1)	99% 99%	LR216277
287_McS4-7	*M. chamomilla* Sekem	MRS microaerobic	3	*Lactobacillus nenjiangensis* (NR_125563.1)	99%	LR216278
280_KA4-12	*B. oleracea* Austria	MRS microaerobic	4	*Lactobacillus paracasei* (NR_041054.1)	99%	LR216279
1_KKS1-1	*B. oleracea* Sekem, small	R2A aerobic	5	*Lactobacillus fabifermentans* (NR_113339.1)	99%	LR216280

^a^Determined by amplified rRNA gene restriction analysis with the restriction endonuclease *Hha*I.

^b^NCBI’s reference RNA sequence database.

^c^Sequences were submitted to the European Nucleotide Archive (www.ebi.ac.uk/ena).

## Discussion

We elucidated lactic acid bacteria in the phyllosphere of the medicinal plants *Matricaria chamomilla* L. and *Calendula officinalis* L. including a natural fermentation of leaves via a time course study. During the fermentation time course, natural phyllospheric LAB were enriched, and different species of *Lactobacillus* and *Enterococcus* could be cultivated from the fermented medicinal plants.

The *Asteraceae* medicinal plants revealed a plant-specific phyllosphere microbiome (Fig. 1), even while grown in close proximity to each other. Accordingly, we suggest that abiotic factors can be excluded for the differences between the microbial compositions within these medicinal plant microbiomes, and that each leaf environment is hosting a unique bacterial composition. Other studies have found this plant specificity for the rhizosphere and root microbiome of many plant species^27,28^, while we present data that the phyllosphere also presents a similar pattern of specificity. The endosphere of both medicinal plants was heavily dominated by *Gammaproteobacteria*, as it was already described for some other endophytic plant communities, e.g., in tomato, lettuce and banana plants^29–31^. *Gammaproteobacteria* were also identified as the most prevalent bacterial class in both medicinal plant ectospheres, making up approximately one-third of the total bacterial colonization. The class of *Bacilli* to which the LAB are belonging was found in a much lower abundance in the original phyllosphere microbiome, but *Lactobacillales* were enriched during the decomposition process of plant material (Fig. 2). In the course of the fermentation, *Gammaproteobacteria* were supplanted by communities belonging to the phylum of *Firmicutes*, besides *Lactobacillales* also *Clostridiales*. Among the LAB, *Enterococcaceae* were identified as the most dominant family in both plants (Fig. 3). The remaining LAB community was remarkably different between the fermentation approaches of the two medicinal plants: *M. chamomilla* revealed higher relative abundances of *Lactobacillaceae* and *Carnobacteriaceae*, while *C. officinalis* showed a higher presence of *Leuconostocaceae* and *Streptococcaceae*. Interestingly, the most abundant family belonging to the *Bacilli* were not LAB but *Planococcaceae*. The genera *Planomicrobium* and *Sporosarcina* of the family of *Planococcaceae* have previously been detected as inhabitants of native Egyptian desert soil^32^. However, in the phyllosphere microbiome of the investigated desert farm plants primarily *Lysinibacillus* and *Planococcus* were detected, whereby especially *Lysinibacillus* demonstrated high tolerance to the acidification during the fermentation process (down to a pH of 2.8). The pH certainly had a significant impact on community dynamics within the fermentation approaches in general. While the pH of the chamomile approach during fermentation with 2.8 was highly acidic, the *Calendula* fermentation reached only a pH of 6.4.

The potential of plant-inhabiting LAB in the preservation of foods and feeds due to their ability of production of antibacterial and antifungal compounds^33–35^ is known since centuries and has been demonstrated by many studies^36^. In the presented study, LAB with leaf ectophytic and endophytic origin from *Asteraceae* medicinal plants were detected and isolated (Table 1), revealing an impressive level of plant-specificity. LAB have previously been ascertained as natural inhabitants of the ectospheric phyllosphere of a broad spectrum of plants, whereby remarkable presence was uncovered in the leaf microbiomes of plants exposed to harsh environmental conditions and grown under organic management, respectively^37,38^ – both true for our sampling sites. An endophytic lifestyle of LAB has been primarily investigated for cereal crops, in their processing they serve as natural inoculum in sourdough fermentation^39^. LAB have also been detected in quite high abundances in the endosphere of Mediterranean olive trees, revealing significantly higher presence in eastern than in western Mediterranean regions^40^. A variety of endophytic LAB has also been detected and cultivated from *Cucurbitaceae* seeds, suggesting edible cucurbit seeds as probiotic food product^41^. However, to our knowledge, medicinal plants have never been investigated in this respect, although a combination of their phytotherapeutic metabolites with a probiotically active microbiome following fermentation could bear a beneficial health effect in two different ways in parallel.

Aside from their function as probiotics and the promotion of human health, LAB also play a role in agriculture by promoting plant health through their antimicrobial potential against several plant pathogens^42,43^. Due to the emerging awareness of the disadvantages of chemical treatment methods, microorganism-based treatments are continuously gaining higher acceptance^44^. Chemical means are not only toxic to humans and the environment, problems occur as well with rising resistances of some pathogens and the high costs associated with the development of new pesticides. Furthermore, some chemicals are not applicable in postharvest treatment^44^. LAB have, for instance, been shown to be able to control the growth of mold during cocoa bean fermentation, enhancing cocoa bean and chocolate quality by reducing off-flavors and mycotoxin contamination. In the study of Ruggirello *et al*.^45^, *Lactobacillus fermentum* and *Lactobacillus plantarum* were among the most promising biocontrol candidates, stemming fungal growth due to substrate competition and the production of antifungal metabolites. Besides LAB use in combating toxin-producing fungi in foods, several LAB do also show potential in the manufacturing of vitamins, thereby competing with the chemical production in terms of sustainability but also economic conditions. Initial cereal-based products are fermented with LAB (e.g., *Lactococcus lactis*, *Streptococcus thermophilus*, *Lactobacillus reuteri*, *Lactobacillus plantarum*) to increase the vitamin B2, B11, and B12 content^46^. Nutrient density is also enhanced by decreasing the sugar content of the starting material, and targeted use of starters in fermentations can, apart from the production of vitamins, hydrolyze anti-nutrients and toxic factors and boost the bioavailability of specific compounds. In the case of the fermentation of legumes, the value of proteins, peptides and amino acids can be increased, making fermented plant-based products an alternative and complement source to animal proteins^5^. These developments have high potential, due to an increasing number of consumers are in search of non-dairy products as a source of probiotics. Reasons therefore are either based on ethical and economic concerns but also on dietary restrictions due to medical reasons, e.g., allergies to milk proteins or lactose intolerance. One way to provide an alternative non-dairy source was documented by Pavli *et al*.^47^ and is based on the incorporation of probiotics into edible polymer matrices used as bioactive packaging material.

The majority of LAB are classified as having GRAS (generally-recognized-as-safe) status, and their application is quite safe, both from a human and environmental point of view^48,49^. However, there are also exceptions: for instance, some species of *Enterococcus* or *Streptococcus* can cause opportunistic infections in humans, whereby the intrinsic resistance of LAB to many antibiotics is an additional risk factor^50,51^. In a recently published WHO list of priority pathogens for research and development of new antibiotics, *Enterococcus faecium* was listed with high and *Streptococcus pneumoniae* with medium priority^52^. The genus *Lactobacillus* is most emphasized in literature for its probiotic and health beneficial activities, as its isolation is reported from a vast diversity of fermented and unfermented sources. However, it was reported that members of the genera *Lactococcus*, *Enterococcus*, *Pediococcus*, and *Leuconostoc* have at least equal potential^36^.

## Conclusions

Our data presented showcase significant microbiome shifts in the course of the implemented fermentations of field-grown *Asteraceae* medicinal plants. The dominant group of *Gammaproteobacteria* was gradually supplanted by communities belonging to the phylum of *Firmicutes*, whereby phyllospheric LAB were enriched in all approaches but never dominated. Besides the leaf surfaces, also the leaf endosphere could be identified as a source for biopreservative LAB. The natural leaf microbiome and the indigenous LAB communities were shown to be habitat-specific and plant-specific – before and during the fermentation. Hence, medicinal plants are not only characterized by unique profiles of secondary metabolites but also of microbial colonization, and a combination of their phytotherapeutic properties with a probiotically active microbiome following fermentation could bear a beneficial health effect in two different ways in parallel. Under careful consideration of the safety and antimicrobial spectrum of LAB, they moreover represent an excellent alternative to synthetic chemical pesticides in sustainable agriculture, potentially even combined with an advantageous effect on human and animal consumers. The obtained collection of cultivable *Lactobacillus* and *Enterococcus* species with medicinal plant origin will be investigated in this respect. LAB and their by-products (e.g., bacteriocins) are already applied industrially in the control of food-borne pathogens^53^, and looking towards the future, they will be even more implicated in the food and agricultural sector as well as in the pharmaceutical field.

## Methods

### The sampling of plant material

Leaf material of the medicinal plants *Matricaria chamomilla* L. and *Calendula officinalis* L. was picked in January 2015 on two organically managed desert farms in Egypt: Sekem near Bilbeis (30°25′05″N, 31°38′16″E) and Faiyum Oasis (29°19′24″N, 30°44′49″E). At the time of sampling, both plant species were in the flowering stage. Four independent replicate composite samples consisting of leaves from at least five individual plants were collected from both plant species at each site.

### Sample preparation and fermentation

The leaf microbiomes were studied separately for the ectosphere (outer surfaces) and endosphere (inner tissues)^54,55^. Ectospheric phyllosphere analysis was performed with 5 g of leaves shaken for 5 min with 15 ml 0.85% sterile NaCl solution in 50 ml reaction tubes (Sarstedt, Nümbrecht, Germany). The leaf wash-offs (6 ml) were subsequently centrifuged at 16,000 × g and 4 °C for 20 min, before freezing the resulting pellets at −70 °C. Endospheric phyllosphere analysis was done with 5 g leaves which were sterilized for 5 min in 4% NaOCl while shaking, washed three times with sterile distilled H_2_O, before being ground with 15 ml 0.85% NaCl solution using a mortar, centrifuged (4 ml) and frozen at −70 °C.

For the fermentation, finely chopped leaves of the medicinal plants were put in 500 ml glass jars (two independent fermentation approaches per plant species – one per sampling site) and mixed with 2% w/w NaCl. For related reasons, three independent fermentation approaches with cabbage (*Brassica oleracea* var. *capitata* L.) were performed in parallel. Two cabbage heads originated from the Sekem farm in Egypt, while the third was bought on a farmers market in Austria. Plant material was mashed and weighted down to be pressed tightly and covered with 0.85% NaCl solution ensuring anaerobic conditions. Approaches were incubated at room temperature for six weeks; pH values were measured after the initial three weeks of fermentation (*M. chamomilla*: Sekem 2.8 and Faiyum 3.8; *C. officinalis*: Sekem 6.4 and Faiyum 6.8; *B. oleacea*: Sekem big 2.8, Sekem small 3.0 and Austria 2.7). Samples for metagenomic DNA extraction were taken every week by withdrawing 15 ml of the liquid and 5 g of the fermented plant material. Collected samples were ground with a mortar, and 6 ml of each approach were undergone a centrifugation step (16,000 × g, 20 min, 4 °C) and stored at −70 °C.

DNA of all prepared frozen original and fermented samples was extracted from the gathered pellets employing the FastDNA SPIN Kit for Soil (MP Biomedicals, Solon, OH, USA) following the manufacturer’s protocol.

### 16S rRNA gene profiling by Illumina MiSeq sequencing

The Sekem sampling site was selected for detailed bacterial profiling of the medicinal plant fermentation by 16S rRNA gene amplicon sequencing, which was implemented of fresh samples from the phyllospheric ectospheres and endospheres and of fermented plant samples covering a weekly fermentation period of six weeks. Amplification of the gathered DNA was performed by PCR using the peptide nucleic acid clamps pPNA and mPNA^56^ to block the amplification of the host’s chloroplastic and mitochondrial DNA. The hypervariable V4 region of the 16S rRNA genes was amplified using region-specific primer pairs carrying Illumina cell flow adaptors and sample-specific barcodes. Per sample, a 30 µl approach was prepared to consist of 1 × Taq-&GO (MP Biomedicals, Solon, OH, USA), 0.2 µM barcoded universal primer 515 f, 0.2 µM barcoded universal primer 806r^57^, 0.75 µM of each of the PNAs (PNA Bio, Thousand Oaks, CA, USA) and 2.0 µl template DNA (~1.5 ng). Amplification in a thermocycler (Biometra, Göttingen, Germany) started with a denaturation step at 96 °C for 5 min, followed by 30 cycles of 96 °C, 1 min, 78 °C, 5 s (PNA annealing), 54 °C, 1 min (primer annealing), 74 °C, 1 min and elongation at 74 °C for 10 min. PCR of each sample was done in independent triplicates, which were pooled together (3 × 30 µl) in the cleaning step by employing the Wizard SV Gel and PCR Clean-Up System (Promega, Madison, WI, USA). DNA concentrations were spectrophotometrically determined (NanoDrop 2000c, Thermo Scientific, Waltham, MA, USA), all approaches were pooled together in an equimolar ratio and subjected to Illumina MiSeq sequencing (chemistry v3, 300 bp paired-end) at Eurofins Genomics (Ebersberg, Germany).

Raw sequencing paired-end reads were assembled with default settings of PANDAseq software, version 2.8^58^. Barcode and primer sequences were trimmed by PRINSEQ software, version 0.20.4^59^. Also, low-quality reads defined as reads with an average quality score below 25, with more than one ambiguous base and a length <250 and >260 were removed using PRINSEQ. The processing of filtered reads to operational taxonomic units (OTUs) was done with the LotuS program using UPARSE at 97% similarity and USEARCH in subsequent seed extension^60^. OTUs assigned to plant-derived chloroplasts, mitochondria and archaeal 16S rRNA, as well as singletons and doubletons were filtered from the dataset by QIIME 1.9.1^61^, which was also used for further microbiome and statistical analyses. For alpha and beta diversity analyses, OTU tables were normalized to the same number of quality reads per sample. Statistical analyses for comparing alpha diversity measurements were performed using the nonparametric *t*-test with 999 Monte Carlo permutations. Beta diversity was analyzed based on Bray-Curtis dissimilarities; the adonis test with 999 permutations was used for corresponding statistics. Significant differences at OTU level were ascertained with Metastats^62^, where p values were computed using a combination of the nonparametric *t*-test, exact Fisher’s test, and the false discovery rate with 10^3^ permutations. Venn diagrams were obtained by using Bioinformatics & Evolutionary Genomics software^63^. The heat map was visualized in Heatmapper^64^.

### Cultivation of lactic acid bacteria

After six weeks of fermentation, bacteria were cultivated on MRS and R2A agar containing 20 µg ml^−1^ cycloheximide (preventing fungal growth) at 30 °C under four different cultivation conditions. One approach was incubated aerobically on R2A to obtain a broad spectrum of all cultivable bacteria. Further approaches were performed on MRS agar^65^, which enables luxuriant growth of LAB especially of fastidious, slower-growing types; incubations were done aerobically, anaerobically in a desiccator with AnaeroGen packs (Oxoid, Basingstoke, UK), and microaerobically performing the pour plating method as recommended in the product data sheet (Carl Roth, Karlsruhe, Germany). Colony forming units (log_10_ CFU ml^−1^) were counted for the individual approaches and cultivation conditions. Significant differences were calculated with SPSS Statistics 23 (SPSS Inc., Chicago, IL, USA) using Tukey-HSD and Games-Howell post hoc tests, depending on the homogeneity of variances. Isolates were encoded using a combination of letters and numbers indicating: (1) consecutive number of the isolates, (2) plant species (Mc = *Matricaria chamomilla*, Co = *Calendula officinalis*, K = *Brassica oleracea* var. *capitata* [KG = big cabbage head, KK = small cabbage head]), (3) farm (S = Sekem, F = Faiyum, A = Austria), (4) isolation method (1 = R2A aerobic, 2 = MRS aerobic, 3 = MRS anaerobic, 4 = MRS microaerobic), and (5) consecutive number per set of isolates.

Isolates which could be classified as potential LAB (Gram-positive in the KOH test and catalase-negative) were selected for genotypic characterization. Genomic DNA was extracted following the protocol of Berg *et al*.^66^ modified with an initial mechanical cell disruption step with glass beads in a FastPrep Instrument (MP Biomedicals, Santa Ana, CA, USA; 30 s, 6.5 m s^−1^). Strains showing varying patterns in performed BOX-PCR genomic fingerprints (amplification with the BOXA1R primer^67,68^) and amplified rRNA gene restriction analysis (ARDRA) with the restriction endonuclease *Hha*I (MP Biomedicals, Eschwege, Germany) were selected and subjected to partial 16S rRNA gene sequencing (LGC Genomics, Berlin, Germany) according to Berg *et al*.^66^. Sequences were aligned with the BLAST algorithm against NCBI’s reference RNA sequence database to check their genetic affiliation to the LAB.

## Supplementary information

Supplementary information

## Data Availability

The datasets generated and analyzed during the current study are available in the European Nucleotide Archive (www.ebi.ac.uk/ena) under the BioProject accession number PRJEB15322.
