# Supplementary material for: Deciphering the microbiome shift during fermentation of medicinal plants
Source: Sci Rep. 2019 Sep 17;9:13461. doi: 10.1038/s41598-019-49799-2 (PMC6748931; doi:10.1038/s41598-019-49799-2)
Supplement: Supplementary file 1 — Supplementary information [file 41598_2019_49799_MOESM1_ESM.pdf]

Deciphering the microbiome shift during fermentation of medicinal plants

Martina Köberl[1,+], Sabine Erschen[1,+], Mohammad Etemadi[1], Richard Allen White III[2,3], Tarek F. El-Arabi[4,5], Gabriele Berg[1]

[1]*Graz University of Technology, Institute of Environmental Biotechnology, Graz, Austria*

[2]*Washington State University, Department of Crop and Soil Sciences, Pullman, WA, USA*

[3]*Washington State University, Department of Plant Pathology, Pullman, WA, USA*

[4]*Ain Shams University, Faculty of Agriculture, Cairo, Egypt*

[5]*Heliopolis University, Biotechnology Laboratory, Cairo, Egypt*

[+]these authors contributed equally to this work

Table S1. Observed species (OTUs) and Shannon diversity indices obtained at 97% sequence similarity for rarefied 16S rRNA gene sequence data of original and fermented medicinal plant samples.

Sample[a]	OTUs	Shannon index
Mc1-Ec	136	5.58
Mc2-Ec	229	6.46
Mc3-Ec	115	5.45
Mc4-Ec	180	5.75
Co1-Ec	241	6.69
Co2-Ec	183	5.90
Co3-Ec	208	6.31
Co4-Ec	123	4.84
Mc1-En	83	3.84
Mc2-En	85	3.87
Mc3-En	89	4.00
Mc4-En	87	3.87
Co1-En	86	4.04
Co2-En	84	3.91
Co3-En	89	4.17
Co4-En	85	3.85
Mc-W1	43	2.59
Mc-W2	40	2.25
Mc-W3	65	3.54
Mc-W4	76	4.01
Mc-W5	73	4.36
Mc-W6	74	4.56
Co-W1	50	3.34
Co-W2	55	3.80
Co-W3	68	4.10
Co-W4	60	3.90
Co-W5	62	4.11
Co-W6	60	3.71

[a]Sample abbreviations indicate: (1) Mc = *Matricaria chamomilla*, Co = *Calendula officinalis*, (2) Ec = ectosphere, En = endosphere, W1-W6 = weeks of fermentation.
